# Paternal mosaicism in *ASXL3*-related bainbridge-ropers syndrome: implications for genetic counseling and prenatal diagnosis

**DOI:** 10.3389/fped.2025.1655021

**Published:** 2025-09-04

**Authors:** Bowen Zhao, Fengjuan Ding, Fei Hou, Hua Jin

**Affiliations:** Department of Prenatal Diagnosis, Jinan Maternity and Child Care Hospital Affiliated Shandong First Medical University (Jinan Maternity and Child Care Hospital), Jinan, Shandong, China

**Keywords:** bainbridge-ropers syndrome1, *ASXL3* gene, whole-exome sequencing, paternal mosaicism, prenatal diagnosis

## Abstract

**Objective:**

Bainbridge-Ropers syndrome (BRS) is a neurodevelopmental disorder predominantly caused by pathogenic variants in the *ASXL3* gene, which have been conventionally considered to occur *de novo*. This study aimed to investigate the potential role of parental mosaicism in BRS inheritance and its clinical implications for genetic counseling.

**Methods:**

Trio-based whole-exome sequencing (WES) was performed on the proband and both parents to identify candidate variants, which were subsequently validated by Sanger sequencing. *ASXL3*-targeted ultra-deep sequencing of paternal semen DNA was then carried out to detect low-level mosaicism. Prenatal diagnosis via amniocentesis was used to evaluate transmission of the familial variant.

**Results:**

We definitively diagnosed this family by WES and found the lowest level of paternal mosaicism reported to date, with a peripheral blood variant allele frequency (VAF) of 8.17% and a semen VAF of 15.03%. Prenatal diagnosis at 18 weeks of gestation confirmed that the variant was not detected in this pregnancy.

**Conclusion:**

This study establishes parental chimerism as an important genetic mechanism for *ASXL3*-associated disorders and emphasizes the need for ultrasensitive testing in genetic counseling. The findings redefine genetic risk stratification for BRS and provide a basis for accurate family planning based on high-depth sequencing.

## 1 Introduction

Bainbridge-Ropers syndrome (BRPS)[OMIM #615,485], a rare autosomal dominant neurodevelopmental disorder first identified in 2013 (1), has become a focus of growing research interest.BRPS is caused by heterozygous loss-of-function mutations in *ASXL3* (18q12.1).BRPS is characterized by multisystemic involvement, including (1) neurodevelopmental manifestations (global developmental delay, moderate to severe intellectual disability, autistic traits, language impairment/absent speech, etc.); (2) neuromuscular/nutritional features (hypotonia, feeding difficulties with failure to thrive, etc.); and (3) dysmorphic features (frontal bossing, arched eyebrows, hypertelorism, down slanting palpebral fissures) (2, 3). These characteristic phenotypes facilitate preliminary clinical identification. While the exact prevalence of BRPS is unknown, the Deciphering Developmental Disorders study (*n* = 9,625 ID trios) identified *de novo ASXL3* variants in 50 probands (1:193), placing *ASXL3* among the top 10 most frequently mutated genes in neurodevelopmental disorders (4).

Current literature on this disorder primarily consists of case reports, with most cases caused by *de novo* mutations. Mosaicism in *ASXL3* has emerged as a significant phenomenon in BRPS research, profoundly influencing disease pathogenesis, progression, and inheritance patterns (5). This study presents a detailed characterization of paternally inherited *ASXL3* mosaicism at low variant allele fractions (VAFs) in phenotypically normal fathers leading to BRPS offspring. These findings have critical implications for accurate genetic counseling, informed reproductive decision-making, and prenatal diagnostic strategies.

## 2 Materials and methods

### 2.1 Patients and clinical information

**Figure 1:**
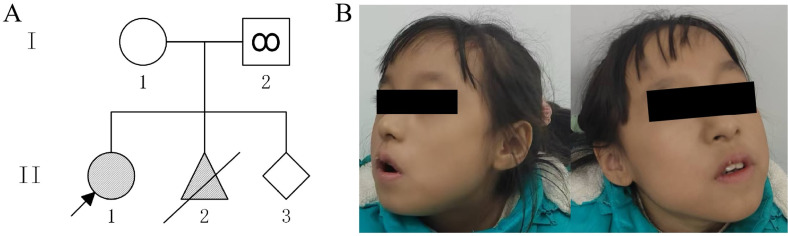
**(A)** Pedigree of the family with genotype results for *ASXL3* (c.1534_1535del, p.Leu512Alafs*4) variant. (I-1) Mother of the proband; (I-2) Father with *ASXL3* (c.1534_1535del, p.Leu512Alafs*4) variant in his sperm; (II-1) Proband; II-2 Pregnancy termination performed due to an ASXL3 (c.1534_1535del, p.Leu512Alafs*4) variant;(II- 3) fetus. **(B)** Clinical characteristics of the proband:global developmental delay,frontal bossing, everted lower lip, dental crowding, hypotonia, feeding difficulties, and autism spectrum disorder.

The proband was an 8-year-old girl (II1) who presented with a global developmental delay characterized by profound speech and motor impairments, accompanied by distinctive craniofacial features (including frontal bossing, everted lower lip, and dental crowding), feeding difficulties, generalized hypotonia, and autism spectrum behaviors (notably poor eye contact and stereotypic hand movements) (Figure 1). She was born at term by vaginal delivery complicated by birth asphyxia (Apgar scores unavailable) and required 10 days of respiratory support in the neonatal intensive care unit. During infancy, she showed failure to thrive, necessitating nutritional supplementation, and her gross-motor milestones were substantially delayed, with head control achieved at 12 months, independent sitting at 18 months, and ambulation at 36 months. At age 3 years, a brain MRI revealed delayed white matter myelination and mild ventriculomegaly. At the current evaluation (8 years), she remained nonverbal, with persistent global delay (height 120 cm, 25th percentile; weight 17.5 kg, <5th percentile). The parents denied consanguinity and reported no significant family history of inherited disorders. The father (I2), a mosaic carrier of the pathogenic variant, underwent comprehensive clinical assessment. No neurodevelopmental abnormalities or facial dysmorphism were observed. The proband's 33-year-old mother(I1), who previously underwent pregnancy termination after detection of an ASXL3 heterozygous variant by external amniocentesis two years ago (II2), is now at 18 weeks’ gestation(II3). She seeks genetic counseling and prenatal diagnostic evaluation to ensure a healthy offspring. First-trimester nuchal translucency ultrasound screening demonstrated normal findings (1.1 mm, within the 50th percentile for gestational age), with no detectable structural anomalies on subsequent detailed fetal anatomical survey.

### 2.2 DNA extraction

Following signing the informed consent, peripheral blood samples (2 ml each) were collected from the proband and biological parents. For the pregnant mother, amniocentesis was performed under ultrasound guidance at 18 weeks of gestation, with 30 ml of amniotic fluid obtained. Genomic DNA was extracted from peripheral blood and amniotic fluid using the MagPure Blood DNA TL Kit (Magen Biotech, China) following the manufacturer's protocol.

### 2.3 Whole-exome sequencing (WES) and pathogenicity analysis

Library preparation and hybridization-based capture were performed, followed by high-throughput sequencing on the NovaSeq 6000 platform (Illumina, San Diego, CA), achieving an average sequencing depth of 200×. Variant annotation and filtering were conducted using public genomic databases, including OMIM (Online Mendelian Inheritance in Man, https://omim.org); GeneReviews (https://www.ncbi.nlm.nih.gov/books/NBK1116/); GHR (Genetics Home Reference, https://ghr.nlm.nih.gov); ClinVar (https://www.ncbi.nlm.nih.gov/clinvar/); HGMD (Human Gene Mutation Database, http://www.hgmd.cf.ac.uk/); PubMed (https://pubmed.ncbi.nlm.nih.gov). The pathogenicity of identified variants was classified according to the American College of Medical Genetics and Genomics (ACMG) guidelines (6).

### 2.4 Sanger sequencing

Sanger sequencing verified that the candidate variant was verified for the proband, the amniotic fluid of the second fetus, and the parents. Primer 3.0 designed the primers with a forward primer sequence -GTGAAGCTCACTACTGGACCAA- and a reverse primer sequence -TGGGCTCTCAGAAGAAAAGGAC-. The PCR products were sequenced on the ABI 3730XL DNA analyzer (Applied Biosystems, Foster City, CA, USA).

### 2.5 Target capture-based deep sequencing

PCR amplification of the father's candidate gene was subsequently performed, and the primers were designed by Primer 3.0 with forward primer sequence -AGGATATCTTGATCCCTGAAGA- and reverse primer sequence-AGTCACAGACTTCTAACTGATCGA-. Subsequently, sequencing was performed on the AmCareSeq-2000 sequencer (AmCare Genomics Lab, Guangzhou, China) with a read length of PEx150. Bioinformatics analysis and annotation were the same as for clinical exome sequencing. The depth of coverage was around 10,000 × per coding base. Variants at each position were reported as percentage values to quantify the change in each base, where the sensitivity of detection of the mutational load of coding region variants was more significant than 0.5%. The sequencing data were visualized using the Integrative Genomics Viewer (IGV) version 2.8.13.

## 3 Results

### 3.1 Clinical genetic findings

**Figure 2:**
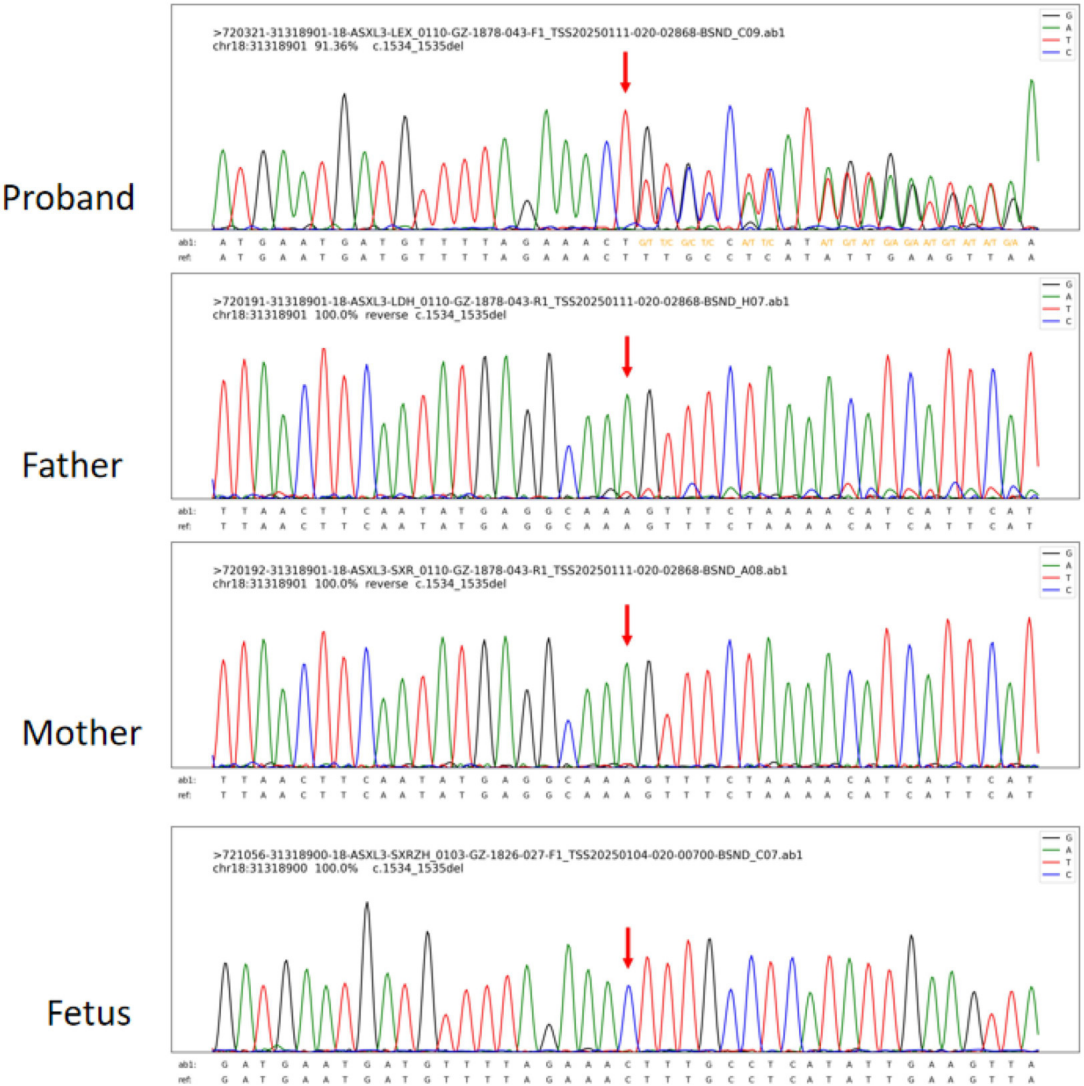
Sanger sequencing results of the proband, the fetus, and their parents [the proband was detected with hemizygous *ASXL3* c.1534_1535del (p.Leu512Alafs*4) variants, while by this method, no variants were detected in the fetus and their parents].

Through integrated phenotype-genotype analysis of clinical exome sequencing data, we identified a heterozygous pathogenic variant in *ASXL3* (c.1534_1535del, p.Leu512Alafs*4) in the proband. Sanger sequencing confirmed this variant in the proband but not in parental samples or amniotic fluid (Figure 2). Given the limited sensitivity of Sanger sequencing for low-level mosaicism, we performed targeted ultra-deep sequencing (10,000× coverage), which detected paternal gonosomal mosaicism at 8.17% VAF in peripheral blood, 15.03% VAF in paternal sperm (Table 1).

**Table 1:** Variant allele frequencies and read counts for parents, Fetus, and proband.

Sample source	Sequencing method	Total depth	Reference supporting reads	Variant supporting reads	Allele frequency (%)
Proband (blood)	Whole- exome sequencing	647	350	297	45.9
Father (blood)		819	750	68	8.17
Mother (blood)		879	878	0	0
Fetus (Amniotic fluid)		683	683	0	0
Father (Semen)	Target capture-based deep sequencing	12,027	12,022	1,807	15.03

### 3.2 Candidate variant pathogenicity ratings

The c.1534_1535del (p.Leu512Alafs*4) variant in *ASXL3* was classified as likely pathogenic based on ACMG/AMP guidelines with the following evidence: (1) PM2-P: the total population variant database frequency of this variant in the database is 0; (2) PVS1: This frameshift variant is predicted to introduce a premature termination codon, potentially triggering nonsense-mediated mRNA decay as it occurs within the terminal exon of all biologically relevant transcripts, thereby meeting PVS1 criteria; (3) PS2-M: confirmed *de novo* occurrence in published cases with matching phenotypes including intellectual disability, language delay, and hypotonia (7) and (4) PS2: paternal mosaicism confirmed by both Sanger sequencing and ultra-deep sequencing (8.17% VAF in blood;15.03% VAF in sperm). This variant meets the criteria for likely pathogenic classification through its combination of population frequency data, predicted molecular consequences, established disease association, and inheritance pattern.

## 4 Discussion

*ASXL3* gene, the largest member of the ASXL gene family (12 exons encoding 2,248 amino acids), orchestrates critical neurodevelopmental processes through its conserved domains (ASXN, ASXH, ASXM1, ASXM2, PHD) (8). Studies demonstrate that *ASXL3* governs the fate specification of the hindbrain, neural crest, and primary neurons during early neurodevelopment. Loss of *ASXL3* function results in aberrant neural plate patterning, phenocopying the neural and craniofacial defects observed in BRS patients (9). Current evidence establishes that *ASXL3* regulates the level of histone H2A lysine 119 monoubiquitination (H2AK119Ub1) through the Polycomb repressive deubiquitination (PR-DUB) complex, directly participating in the transcriptional regulation of brain development genes (such as HOX gene clusters) and neurodevelopmental pathways. Loss of *ASXL3* function leads to abnormal chromatin states and dysregulation of key gene expression, ultimately causing structural and functional brain defects, which provides a molecular mechanism for neurodevelopmental disorders such as BRS (10).

The previously reported *ASXL3* gene variants were predominantly *de novo* mutations, with only speculative mentions of mosaicism. This study presents the first comprehensive analysis of *ASXL3* mosaicism (Table 2). Koboldt et al. (11) first proposed germline mosaicism as a potential mechanism for familial BRPS through their report of affected sisters sharing an identical *de novo* nonsense variant in *ASXL3* (p.Tyr392*). Schirwani (12) and colleagues subsequently identified five affected individuals across three families, with two families exhibiting germline mosaicism patterns. Their cohort included an 11-year-old female presenting with hypotonia, feeding difficulties, intellectual disability, global developmental delay, and behavioral abnormalities. WES demonstrated 30%–35% VAF in matched blood and saliva samples. In their 2021 follow-up study, Schirwani's team characterized three inheritance patterns: the p.(Gln1512*) in P2 and the p.(Gln931fs) in P10 were inherited from the affected parents, and the p.(Leu1481fs) in P22 was inherited from the apparently asymptomatic but highly educated mother. No chimerism was detected in the blood samples tested. However, chimerism in other tissues could not be excluded (7). Additionally, a recent study reported a novel case involving two non-twin siblings carrying a likely pathogenic variant. This variant was inherited from an unaffected parent who exhibited mosaicism, which was detected in approximately 30% of the analyzed cells in peripheral blood DNA samples (5). Our study reports the lowest-level paternal mosaicism documented to date in BRPS (8.17% VAF in peripheral blood; 15.03% VAF in sperm). Prenatal diagnosis via amniocentesis at 18 gestational weeks yielded negative results.

**Table 2:** Summary of clinical cases of presumed *ASXL3* gene mosaicism.

Patients (family ID)	Gender	Age	Samples	Gene	Genetic variant	Mosaicism type/detection method	Mosaicism level	Main clinical features
1 (the proband, this study)	F	8 y	Blood	*ASXL3*	c.1534_1535del (p.Leu512Alafs*4)	*de novo* (trio-WES); low-level paternal mosaicism (ultra-deep sequencing)	Father: 8.17% (blood), 15.03% (semen)	GDD, absent speech (nonverbal), growth retardation (height 25th percentile; weight <5th percentile)
2 [P1, (Koboldt et al., 2018)]	F	16 y	Blood	*ASXL3*	c.3106C > T (p.R1036X)	Suspected gonadal mosaicism (parental Sanger negative)	/	Severe ID, absent speech, epilepsy, craniofacial anomalies, corpus callosum hypoplasia
3 [P2, (Koboldt et al., 2018)]	F	15 y	Blood	*ASXL3*	c.3106C > T (p.R1036X)	Suspected gonadal mosaicism (parental Sanger negative)	/	Same as P1
4 [P8, (Fu et al., 2019)]	F	7 m	Blood	*ASXL3*	c.4330C > T (p.Arg1444)	Suspected gonadal mosaicism (maternal Sanger negative; paternal untested)	/	Congenital hypothyroidism, DD, hypotonia
5 [Family 1 (P1), (Schirwani et al., 2020)]	F	12 y	Blood, saliva	*ASXL3*	c.3284_3288del (p.Thr1096AsnfsTer12)	Gonadal mosaicism (parental saliva negative)	/	Severe DD, hypotonia, dysmorphic features, feeding difficulty, autism, hand flapping
6 [Family 1 (P2), (Schirwani et al., 2020)]	M	5 y	Blood, saliva	*ASXL3*	c.3284_3288del (p.Thr1096AsnfsTer12)	Gonadal mosaicism (parental saliva negative)	/	Severe DD, hypotonia, dysmorphic features, feeding difficulty, cleft palate, clubfoot, recurrent ear infections
7 [Family 2 (P3), (Schirwani et al., 2020)]	M	5 y	Blood	*ASXL3*	c.4509_4513dup (p.Val1505AspfsX3)	Gonadal mosaicism (parental blood negative)	/	Feeding difficulty, hypotonia, DD, language impairment, ear infections
8 [Family 2 (P4), (Schirwani et al., 2020)]	M	2 y	Blood	*ASXL3*	c.4509_4513dup (p.Val1505AspfsX3)	Gonadal mosaicism (parental blood negative)	/	Autistic features, self-injury, feeding difficulty, sleep apnea
9 [Family 3 (P5), (Schirwani et al., 2020)]	F	11 y	Blood, saliva	*ASXL3*	c.1632_1637delins31 (p.Pro545LeufsTer10)	Postzygotic mosaicism (ultra-deep sequencing)	30%-35%	Severe delay, no speech, hypotonia, dysmorphic features, self-injury, seizure
10 [P22, (Schirwani et al., 2021)]	/	/	Blood	*ASXL3*	c.4441dup (p.Leu1481fs)	Inherited from asymptomatic mother (non-blood mosaicism possible)	/	Not detailed; cohort: ID, delay, speech loss, feeding issues, hypotonia, dysmorphic features
11 [P7, (Trujillano et al.)]	M	5 y	Blood	*ASXL3*	c.4310T > A (p.Leu1437Ter)	Gonadal mosaicism (father blood VAF 30%)	30% (father)	Mild ID, delay, hypotonia, speech delay, autistic traits, aggression
12 [P15, (Trujillano et al.)]	M	11 y	Blood	*ASXL3*	c.4310T > A (p.Leu1437Ter)	Gonadal mosaicism (father blood VAF 30%)	30% (father)	Severe ID, no speech, hypotonia, autism spectrum, hand flapping

F, female; M, male; y, years; m, months; GDD, global developmental delay; DD, developmental delay; ID, intellectual disability; VAF, variant allele frequency; WES, whole-exome sequencing; NA, not available or not applicable.

Although sperm mosaicism has minimal impact on sperm function, the offspring of affected individuals can inherit germline mutations, which frequently result in severe genetic disorders. Sperm mosaicism represents a major source of *de novo* mutations (DNMs) (13). The distribution of mosaic mutations is largely determined by the developmental timing of the mutational event: (1) Early mutations (pre-gastrulation, 2–4 cell stage): These mutations may propagate into multiple tissues, including germ cells, leading to gonadal-somatic mosaicism. (2) Mid-stage mutations (post-gastrulation, pre-organogenesis): Such mutations are typically restricted to tissues derived from a specific germ layer. (3) Late-stage mutations (post-organogenesis): These usually result in single-organ-specific mosaicism (or chimerism) (14–16). Sperm mosaicism can be divided into three types: Type I arises during sperm meiosis and is nonage dependent; Type II arises in spermatogonia and increases as men age;and Type III arises during paternal embryogenesis, spreads throughout the body, and contributes stably to sperm throughout life. Where Types I and II confer little risk of recurrence, Type III may confer identifiable risk to future offspring.These mutations are likely to be the single largest contributor to human genetic diversity (17). The underlying reason for the predominance of paternal mutation is attributable to the fact that oocytes undergo prolonged arrest following meiosis, while spermatogonial stem cells undergo continuous mitosis throughout their lifespan. This results in a significantly higher accumulation of replication errors and DNA damage in spermatogonial stem cells during division compared to oocytes. During the process of embryonic development, cells that carry different mutations exhibit varying capabilities with respect to survival and proliferation. Cell competition plays a pivotal role in embryonic development, influencing the distribution and proportion of mutated cells across different tissues. This, in turn, affects embryonic development and the onset and progression of diseases. The process of cell competition is generally accomplished via several different pathways, including apoptosis, proliferation regulation, mitochondrial function, and the dynamics of stem cells and progenitor cells (18).

Mosaicism complicates the inheritance pattern of genetic disorders in genetic counseling, posing challenges for accurate recurrence risk assessment. Conventional sequencing approaches often fail to detect mosaicvariants, necessitating the integration of high-depth sequencing and genetic pattern analysis for precise risk evaluation. The identification of mosaicism has broadened the genetic spectrum of BRPS, demonstrating that overlooking the possibility of gonadal mosaicism may lead to underestimation of disease recurrence risk in familial cases. The presence of mosaicism underscores the need for heightened clinical vigilance, particularly in multiplex families, and the implementation of more comprehensive and precise testing strategies to accurately characterize genetic variants.

In genetic counseling practice, potential mosaicism must be thoroughly considered. Parents should undergo detailed genetic testing and evaluation to ensure accurate risk stratification and reproductive guidance, enabling informed decision-making. While this family pursued naturally conceived pregnancy with prenatal diagnosis, preimplantation genetic testing (PGT-M) could have enabled earlier selection of unaffected embryos (19). Future studies should further elucidate the mechanisms underlying mosaicism and its phenotypic modulation, providing a foundation for personalized medicine.

In conclusion, this study significantly advances our understanding of *ASXL3*-related disorders by systematically characterizing paternal mosaicism in BRS, reporting the lowest-level germline mosaicism to date (8.17% VAF in peripheral blood; 15.03% VAF in sperm). This work redefines the genetic architecture of BRS, emphasizing that comprehensive parental testing and advanced sequencing technologies are indispensable for precise genetic counseling and family planning.

## Data Availability

The datasets presented in this study can be found in online repositories. The names of the repository/repositories and accession number(s) can be found in the article/Supplementary Material.

## References

[1] Bainbridge MN Hu H Muzny DM Musante L Lupski JR Graham BH *de novo* truncating mutations in asxl3 are associated with a novel clinical phenotype with similarities to bohring-opitz syndrome. Genome Med. (2013) 5(2):11. 10.1186/gm415 23383720 PMC3707024

[2] Woods E Holmes N Albaba S Evans IR Balasubramanian M. Asxl3-related disorder: molecular phenotyping and comprehensive review providing insights into disease mechanism. Clin Genet. (2024) 105(5):470–87. 10.1111/cge.14506 38420660

[3] Balasubramanian M Schirwani S. ASXL3-Related disorder. In: Adam MP Feldman J Mirzaa GM Pagon RA Wallace SE Amemiya A, editors. GeneReviews®. Seattle, WA: University of Washington, Seattle (2020). 33151654

[4] Wright CF Fitzgerald TW Jones WD Clayton S McRae JF van Kogelenberg M Genetic diagnosis of developmental disorders in the ddd study: a scalable analysis of genome-wide research data. Lancet. (2015) 385(9975):1305–14. 10.1016/S0140-6736(14)61705-0 25529582 PMC4392068

[5] Trujillano L Valenzuela I Costa-Roger M Cusco I Fernandez-Alvarez P Cueto-Gonzalez A Comprehensive clinical and genetic characterization of a Spanish cohort of 22 patients with bainbridge-ropers syndrome. Clin Genet. (2025) 107(6):646–62. 10.1111/cge.14701 39833101

[6] Richards S Aziz N Bale S Bick D Das S Gastier-Foster J Standards and guidelines for the interpretation of sequence variants: a joint consensus recommendation of the American college of medical genetics and genomics and the association for molecular pathology. Genet Med. (2015) 17(5):405–24. 10.1038/gim.2015.30 25741868 PMC4544753

[7] Schirwani S Albaba S Carere DA Guillen SM Milan ZF Si Y Expanding the phenotype of ASXL3-related syndrome: a comprehensive description of 45 unpublished individuals with inherited and *de novo* pathogenic variants in asxl3. Am J Med Genet A. (2021) 185(11):3446–58. 10.1002/ajmg.a.62465 34436830

[8] Katoh M Katoh M. Identification and characterization of ASXL3 gene in silico. Int J Oncol. (2004) 24(6):1617–22. 15138607

[9] Lichtig H Artamonov A Polevoy H Reid CD Bielas SL Frank D. Modeling bainbridge-ropers syndrome in xenopus laevis embryos. Front Physiol. (2020) 11:75. 10.3389/fphys.2020.00075 32132929 PMC7040374

[10] Srivastava A Ritesh KC Tsan YC Liao R Su F Cao X *de novo* dominant asxl3 mutations alter h2a deubiquitination and transcription in bainbridge-ropers syndrome. Hum Mol Genet. (2016) 25(3):597–608. 10.1093/hmg/ddv499 26647312 PMC4731023

[11] Koboldt DC Mihalic Mosher T Kelly BJ Sites E Bartholomew D Hickey SE A *de novo* nonsense mutation in ASXL3 shared by siblings with Bainbridge-ropers syndrome. Cold Spring Harb Mol Case Stud. (2018) 4(3):a002410. 10.1101/mcs.a002410 29305346 PMC5983172

[12] Schirwani S Hauser N Platt A Punj S Prescott K Canham N Mosaicism in asxl3-related syndrome: description of five patients from three families. Eur J Med Genet. (2020) 63(6):103925. 10.1016/j.ejmg.2020.103925 32240826

[13] Jonsson H Sulem P Arnadottir GA Palsson G Eggertsson HP Kristmundsdottir S Multiple transmissions of *de novo* mutations in families. Nat Genet. (2018) 50(12):1674–80. 10.1038/s41588-018-0259-9 30397338

[14] Aluri J Cooper MA. Genetic mosaicism as a cause of inborn errors of immunity. J Clin Immunol. (2021) 41(4):718–28. 10.1007/s10875-021-01037-z 33864184 PMC8068627

[15] Campbell IM Shaw CA Stankiewicz P Lupski JR. Somatic mosaicism: implications for disease and transmission genetics. Trends Genet. (2015) 31(7):382–92. 10.1016/j.tig.2015.03.013 25910407 PMC4490042

[16] Martinez-Glez V Tenorio J Nevado J Gordo G Rodriguez-Laguna L Feito M A six-attribute classification of genetic mosaicism. Genet Med. (2020) 22(11):1743–57. 10.1038/s41436-020-0877-3 32661356 PMC8581815

[17] Breuss MW Yang X Gleeson JG. Sperm mosaicism: implications for genomic diversity and disease. Trends Genet. (2021) 37(10):890–902. 10.1016/j.tig.2021.05.007 34158173 PMC9484299

[18] Waldvogel SM Posey JE Goodell MA. Human embryonic genetic mosaicism and its effects on development and disease. Nat Rev Genet. (2024) 25(10):698–714. 10.1038/s41576-024-00715-z 38605218 PMC11408116

[19] Pan J Li J Chen S Xu C Huang H Jin L. Living birth following preimplantation genetic testing for monogenic disorders to prevent low-level germline mosaicism related nicolaides-baraitser syndrome. Front Genet. (2022) 13:989041. 10.3389/fgene.2022.989041 36160002 PMC9500527

